# Complementary and alternative medicine use in adolescents with inflammatory bowel disease and juvenile idiopathic arthritis

**DOI:** 10.1186/1472-6882-14-124

**Published:** 2014-04-04

**Authors:** Pauliina Nousiainen, Laura Merras-Salmio, Kristiina Aalto, Kaija-Leena Kolho

**Affiliations:** 1 Department of Pediatrics, Kuopio University Hospital and University of Helsinki, Kuopio, Finland; 2 Children's Hospital, Helsinki University Central Hospital and University of Helsinki, P.O. Box 281, Helsinki, Finland

**Keywords:** Children, Colitis ulcerative, Crohn’s disease, Juvenile idiopathic arthritis, Paediatric

## Abstract

**Background:**

The use of complementary alternative medicine (CAM) is potentially prevalent among paediatric patients with chronic diseases but with variable rates among different age groups, diseases and countries. There are no recent reports on CAM use among paediatric patients with inflammatory bowel disease (IBD) and juvenile idiopathic arthritis (JIA) in Europe. We hypothesized that CAM use associates with a more severe disease in paediatric IBD and JIA.

**Methods:**

A cross-sectional questionnaire study among adolescent outpatients with IBD and JIA addressing the frequency and type of CAM use during the past year. The patients were recruited at the Children’s Hospital, University of Helsinki, Finland.

**Results:**

Of the 147 respondents, 97 had IBD (Crohn’s disease: n = 46; median age 15.5, disease duration 3.4 years) and 50 had JIA (median age 13.8, disease duration 6.9 years). During the past 12 months, 48% regularly used CAM while 81% reported occasional CAM use. Compared to patients with JIA, the use of CAM in IBD patients tended to be more frequent. The most commonly used CAM included probiotics, multivitamins, and mineral and trace element supplements. Self-imposed dietary restrictions were common, involving 27.6% of the non-CAM users but 64.8% of all CAM users. Disease activity was associated with CAM use in JIA but not in IBD.

**Conclusions:**

CAM use is frequent among adolescents with IBD and JIA and associates with self-imposed dietary restrictions. Reassuringly, adherence to disease modifying drugs is good in young CAM users. In JIA, patients with active disease used more frequently CAM than patients with inactive disease. As CAM use is frequent, physicians should familiarise themselves with the basic concepts of CAM. The potential pharmacological interaction or the toxicity of certain CAM products warrants awareness and hence physicians should actively ask their patients about CAM use.

## Background

Inflammatory bowel disease (IBD) incidence is on the rise, especially in Western countries [1]. In Finland, the incidence of paediatric IBD is high, approximating 15/100 000 in 2003, with an estimated annual increase of 6.5% [2]. IBD is typically diagnosed in late adolescence or early adulthood and often leads to an impairment of the quality of life [3,4]. Adolescents may suffer from emotional and social problems and have impaired competence compared to their peers [5,6]. Active IBD may also impair cognitive functions [7]. With Crohn’s disease (CD) in particular, adolescents also face the risk of delayed growth and delayed puberty. Since paediatric patients often suffer from more extensive and aggressive forms of the disease, the need for corticosteroids, immunosuppressants and biologics are frequently indicated [8,9]. However, complete remission is infrequent and most patients experience a relapsing disease course.

The incidence of juvenile idiopathic arthritis (JIA), another common chronic autoimmune disease in childhood, is approximately 20-25/100 00 in Finland, but in contrast to IBD, no increase in the number of cases has been observed in recent years [10]. One third of the JIA patients have initially oligoarticular disease without disease progression and a remarkable degree of disability [11]. One-third of JIA patients have polyarticular disease with a high risk of disease progression and disability [12]. The remaining one-third of the patients belongs to the extended oligoarticular, spodylarthropathy or systemic onset JIA group, with a potentially complicated course of disease. Similar to IBD, most patients have repeated relapses [13]. The first-line medications used for JIA are non-steroidal anti-inflammatory drugs (NSAID) and intra-articular corticoid injections, followed by disease modifying drugs (DMARD), and in the most severe cases, new biological drugs [14].

The use of complementary and alternative medicine (CAM) may be frequent among patients with chronic diseases, such as IBD and JIA [15,16], but no recent studies on paediatric and adolescent CAM use have been conducted in the Scandinavian countries and the number of studies worldwide is limited. The concept of CAM includes various medical practices and products that are not commonly considered to be part of conventional medicine. CAM practices are often grouped into five categories: 1) whole medical systems (e.g. homeopathic medicine, acupuncture), 2) mind-body medicine (e.g. meditation, mental healing, hypnosis), 3) biologically-based practices (e.g. herbal products, dietary supplements), 4) manipulative and body-based practices (e.g. chiropractic, osteopathy, massage) and 5) energy medicine (e.g. light therapy, reiki, healing touch) [17,18]. Using CAM therapies may be beneficial, but such therapies can also interact with the conventional medications. Most alarmingly, some products may have serious adverse effects resulting in, for example, a deterioration of the liver function [19].

In paediatric IBD, the frequency of CAM use among patients varies from 6.7% in Canada [20] to estimates of 37% in Scotland [21], 50% in the USA [22-24] and 72% in Australia [25]. In the Nordic countries, an adult IBD study reported that only 7.5% of patients regularly use CAM [15]. With respect to JIA, the frequency of CAM use seems comparable to IBD, at 34–92% [16,26-29]. However, most paediatric studies have only included a limited number of patients and the findings have rarely been related to disease activity. Also, the definition of CAM varies, with some studies including the use of specific diets or regular multivitamin tablets as forms of CAM.

Since CAM use is potentially prevalent among patients with chronic diseases, we assessed the frequency of CAM use in Finnish adolescent IBD and JIA populations. We hypothesized that CAM use associates with a more severe disease in paediatric IBD and JIA populations.

## Methods

### Questionnaire

The use of CAM was assessed using a questionnaire that included 43 items (Additional file 1). The comprehensiveness of the questions was pre-tested in a group of 10 adolescents with no chronic disease. We then handed out the questionnaire to consecutive patients with IBD and JIA, aged 12 to 18 years, during routine follow-up visits at the outpatient clinic of Children’s Hospital, University of Helsinki, a tertiary care hospital, between June 2011 and June 2012. The patients were advised to fill in the form at home together with the primary caregiver and to return it in a prepaid envelope. An additional 100 questionnaires with return envelopes were mailed to patients who did not have scheduled visits during the study period (50 IBD and 50 JIA patients; mailing 1 June 2012). No reminders were sent. During clinical visits, the clinical disease activity was scored according to the physicians’ global assessment (PGA (4)). IBD patients also received a questionnaire assessing disease activity and general wellbeing and JIA patients either completed the Child Health Assessment Questionnaire (CHAQ) (scores 0–3 (29)) and assessed their general wellbeing using a visual analogue scale (VAS, scores 0–100) or else the disease activity was assessed using the modified JADAS10 without the patient’s VAS [30]. All of the questionnaires were coded and analysed anonymously. Exclusion criteria included hospitalized patients.

The questionnaire included items pertaining to the use of products containing vitamins, trace elements, probiotics, energy drinks, recovery products and specific nutritional products for athletes during the past 12 months. Twenty-eight specific questions requested information about whether or not the patient had used a certain group of products or therapy; if they answered “yes”, they were asked a specific follow-up question about the type of product/therapy and how much and how often they had used it. A product used three times a week or more was often considered to fit a pattern of regular use. An open-ended question asked about the use of any other product or therapy not listed. Also, data on the use of special diets or the avoidance of any food ingredients and information on socioeconomic factors (such as family size and housing details) were requested. Furthermore, questions on the use of the medications to treat IBD, JIA or other possible concomitant disease as well as any antibiotics were also included. Adherence to disease-modifying medication was estimated by asking patients how often they forgot to intake the medication prescribed by the physician (monthly or less/weekly/daily). We abstracted this information from the patient charts, the specific data on prescribed IBD/JIA medications, including steroids, immunosuppressive or immunomodulative agents, TNFα-blockers or other biologics, and other IBD/JIA medications (5-aminosalisylic acid/salazopyrine, or hydroxychloroquine) at the time that the patients took part in the study.

In this study, CAM use was defined based on the study by Hilsden et al. [18]. Vitamin D substitution was not considered to be a part of CAM, since Finnish national guidelines (as established by the National Institute of Health and Welfare) support the daily use of 7.5 to 10 μg/day for all children less than 18 years of age. Likewise, since the use of iron products and calcium supplements is usually by prescription, and although data were requested on their use, these products were not considered to be a part of CAM either. Here, we listed probiotics as CAM, even though their possible benefit in IBD is still a point of discussion. While dietary modifications were not treated as CAM in this study, they are discussed separately.

For the group of patients recruited during routine clinical visits, their vitamin D serum levels (D-25-OH-vitamin) and blood count were assessed in the clinical laboratory.

### Ethical considerations

The ethical committee of Helsinki University Hospital approved the study protocol. The patients and/or their guardians who were recruited during clinical visits and agreed to blood sampling signed an informed consent form.

### Statistical analyses

Data are presented either as means (± standard deviation) or as medians (interquartile range), as appropriate for the distribution normality. Correlations between individual continuous parameters were sought using the Spearman correlation and between dichotomous parameters using the Kruskall-Wallis or Mann Whitney U test, when appropriate. Fisher’s exact two-sided test was used for comparisons related to frequencies. GraphPad Prism® Version 5.0c was used for the analyses. The statistical significance was set as p < 0.05.

## Results

The background data for the 147 respondents is shown in Table 1. The total response rate of IBD patients compared to JIA patients was significantly higher (76% versus 51%, p = 0.0001). Of the patients recruited during in-house visits, 8.8% refused to participate in the study (IBD: n = 5, male: n = 2; JIA: n = 8, male: n = 4). The response rates for the IBD and JIA groups who received the questionnaire by mail were 46% and 20%, respectively, comprising in total 34 respondents from the “mailed” group. There were no major differences in the characteristics between the groups recruited as a result of their clinical visit or by mail, so the data have been pooled collectively. The questionnaires were generally completed in full, with missing items on average being less than one per questionnaire.

**Table 1:** Background characteristics of the adolescent patients with inflammatory bowel disease (IBD) and juvenile idiopathic arthritis (JIA)

	**IBD**	**JIA**	**Total**
No. of respondents	97	50	147
% of all respondents	66%	34%	n.a.
(% of recruited patients)	(76%*)	(51%*)	(65%)
Recruited during	74	40	113
Clinical visits (% of respondents)	(76%)	(80%)	(77%)
Recruited by mail	24	10	34
(% of respondents)	(24%)	(20%)	(23%)
Age, median (IQR)	15.5*	13.8*	14.7
	(13.7-16.7)	(12.7-14.9)	(13.0-16.2)
Gender, male	51	17	68
(%)	(53%)	(34%)	(46%)
Disease duration	3.4*	6.9*	3.8
(years), median (IQR)	(1.3-5.4)	(2.7-10.1)	(1.6-7.6)
Disease subtype	Crohn’s: n = 46	Oligoarthritis: n = 18	
	Ulcerative colitis: n = 41	Polyarthritis: n = 25**	
		Enthesitis related: n = 4	
	Unclassified colitis: n = 10	Psoriasis related: n = 3	

IQR = interquartile range; n.a. = not applicable.

*p < 0.001 between IBD and JIA groups.

**including oligoarthritis extended: n = 7.

The IBD patients were somewhat older and with a shorter disease duration compared to the JIA patients (Table 1). We performed a dropout analysis to assess possible selection bias (differences between respondents and non-respondents). Those patients who returned the questionnaire were comparable by age and gender with those who did not return it (IBD non-respondents: n = 27, median age 15.8, male: n = 15; JIA non-respondents: n = 41, median age 14.8, male: n = 15), and based on the chart review, there were no major differences in disease characteristics (data not shown).

During the past year, 48% of all patients regularly used CAM (54% and 38% of IBD and JIA patients, respectively; p = 0.08; Table 2). However, the proportion of all patients who have tried at least once CAM or who have used it irregularly (less than three times a week) was 81%. The most frequently used CAM were probiotics, multivitamin products, and mineral and trace element supplements (Table 2). The mind-body medicine and body-based practices included acupuncture, reflexology, and massage.

**Table 2:** The use of products considered complementary alternative medicine (CAM) among adolescents with inflammatory bowel disease (IBD, n = 97) and juvenile idiopathic arthritis (JIA, n = 50)

	**IBD patients using CAM**	**JIA patients using CAM**
**No. of patients using CAM (percentage of respondents with the given diagnosis)**	**52 (54%)**	**19 (38%)**
	**The proportions of the products used that are considered CAM (among CAM users)**	
Probiotics	62%	58%
Multivitamin products	54%	42%
Minerals and trace elements*	37%	21%
Omega-3 and −6 products	23%	37%
Supplementary nutrition products for athletes	12%	0%
Mind-body medicine	7.7%	0%
Body-based practices and energy medicine	3.8%	5.3%

The differences between the IBD and JIA groups are not statistically significant.

*iron and calcium substitutions excluded.

Of all CAM users, 64.8% consumed special diets, which was significantly higher than for the non-users (27.6%, p < 0.0001). When those patients on a lactose-free diet were excluded (since the diet may be considered to reflect genetic hypolactasia in the study population (31), the respective figures were 48.5% and 13.1% (p < 0.001). The most commonly consumed diets included non-dairy or gluten-free diets and the avoidance of fruits and vegetables, with the frequencies being comparable between IBD and JIA patients (Table 3). CAM users with self-imposed dietary restrictions more often used a combination of several CAM products than those on regular diets (p < 0.0016).

**Table 3:** Diet restrictions related to the use of complementary alternative medicine (CAM) in adolescents with inflammatory bowel disease (IBD: n = 97) and juvenile idiopathic arthritis (JIA: n = 50)

	**IBD patients using a special diet**		**JIA patients using a special diet**		**Total study group (n = 147)**	
**No. of patients with a special diet (percentage of total respondents)**	53 (54.6%)*		14 (28%)*		**67 (45.6%)**	
	**CAM users**	**Non-users**	**CAM users**	**Non-users**	**CAM users**	**Non-users**
	**n = 52**	**n = 45**	**n = 19**	**n = 31**	**n = 71**	**n = 76**
No. of patients with special diets	36 (69.2%)	17 (37.8%)	10 (52.6%)	4 (12.9%)	46 (64.8%)**	21 (27.6%)**
Non-dairy (% of the patients on a special diet)	30.6%	23.5%	10%	25%	26%	23.8%
Lactose-free (% of the patients on a special diet)	22.2%	47.1%	40%	75%	26%	52.4%
Gluten-free (% of the patients on a special diet)	19.4%	11.8%	10%	0%	17.4%	9.5%
Avoidance of fruits/vegetables (% of the patients on a special diet)	27.8%	17.6%	40%	0%	37%	14.3%

*difference between IBD and JIA patients: p < 0.002; **difference between CAM users and non-users: p < 0.0001.

The type of medication used for disease control did not associate with CAM use. Those patients with a moderate to severe disease treated with biologics, corticoids or immunosuppressants used CAM in a comparable manner as the other patients. Also, the proportion of patients reporting poor compliance to the prescribed medication was similar between CAM users and non-users, and comparable between the IBD (19%, with 18/97 reporting weekly non-compliance) and JIA groups (26%, with 13/50 reporting weekly or daily non-compliance, p > 0.05, Fisher’s exact two-sided test). There were no significant gender differences (55% of all CAM users were boys) or differences related to the environment or family (place of residence, family size, domestic animals or pets, smoking) associated with CAM use (data not shown). We found no association between disease duration and CAM use.

We found that among IBD patients, CAM use was not related to disease activity (Table 4). The disease was clinically active in 18% of the IBD patients, with the others displaying clinical remission or only mild activity. Within the group with the most active disease, 59% of the patients reported CAM use as opposed to 44% of the patients with the mildest disease. For JIA patients, the overall disease activity was fairly low and only 24% had JADAS10 scores indicating active disease (high score 3: n = 1, score 2: n = 3, score 1: n = 8; all of the other patients had a score of 0 [30]. Compared to those with minor symptoms, CAM use among JIA patients with active disease was more frequent (p = 0.038, Table 4).

**Table 4:** Disease activity and the use of complementary alternative medicine (CAM) in adolescents with inflammatory bowel disease (IBD) and juvenile idiopathic arthritis (JIA)

	IBD (n = 95)*		JIA (n = 50)**	
	**CAM users**	**Non-users**	**CAM users**	**Non-users**
	**n = 45**	**n = 50**	**n = 19**	**n = 31**
Inactive or mild disease activity	25	28	11	27
Moderate to severe disease activity	22	22	8	4

*data missing: n = 2; **CAM significantly more frequent among patients with active JIA (p < 0.05).

We found no difference in haemoglobin levels when it came to CAM use (median levels 128 g/l and 125 g/l in users and non-users, respectively). Likewise, D-25-OH levels in serum were comparable between CAM users and non-users (median 77 nmol/l IQR 56–97 and 75 nmol/l, IQR 56–88, respectively). Neither were there significant differences when these parameters were compared between the IBD and JIA groups (data not shown).

## Discussion

This cross-sectional questionnaire study assessed CAM use in adolescent IBD and JIA patients. We found that CAM use was frequent. During the preceding 12 months, almost every other patient had regularly used CAM while the proportion of patients reporting at least occasional CAM use was 81%. For IBD patients, the use of CAM seemed more frequent than for JIA patients. Unexpectedly, dietary restrictions were common and almost two-thirds of all CAM users reported that they were on special diets. Disease activity did not associate with CAM use in IBD patients, whereas JIA patients with more active disease used CAM more frequently. However, the small number of JIA patients may bias the results. Reassuringly, compliance with disease-modifying drugs was not affected by CAM use.

CAM remains a field of uncertainty for many practitioners [18]. It is not easy to define CAM and the definitions vary between countries and cultures: in Asian countries, acupuncture is usually not considered a form of CAM, as it would be, for instance, in the Scandinavian countries. As fish oils and probiotics are widely used, some researchers no longer consider them CAM. We included probiotics within the concept of CAM, with them being the most frequently used form of CAM followed by multivitamin products, mineral and trace element supplements, and omega-3 and −6 products. On the other hand, we did not consider vitamin D substitution or the use of iron or calcium supplementation as CAM. Here, the patients’ D-25-OH and haemoglobin levels were well within the given cut-off for normal values regardless of whether or not they reported using CAM.

Chronic disease has been reported to associate with CAM use in children [31], but research on paediatric patients is limited. In North America, CAM use among both paediatric IBD [22-24] and JIA patients [16,26-29] is considerably more common than among healthy children ([32]. The most commonly reported forms of CAM use among children with IBD included proiotics, fish oils, herbs, dietary modifications and megavitamin therapy, all of which are well in line with our findings. Here, the regular use of CAM was within the range reported in earlier studies, but we found that a higher proportion of the patients (81%) use CAM at least occasionally.

Unexpectedly, 65% of CAM users reported various dietary modifications, including a gluten-free diet and the avoidance of fruit and vegetables. A Scottish study found that 28% of patients had adopted a dairy-free diet as a dietary modification, which corresponds to the proportion of dietary modifications reported by non-CAM users in our study [21]. Perhaps surprisingly, JIA patients also reported following special diets, with there being no significant differences compared to the IBD patients. Interestingly, compared to patients on regular diets, those with self-imposed restricted diets also used multiple types of CAM significantly more often. This association most likely reflects the attitudes of the caregivers to CAM, but it still warrants a thorough history taking and nutritional assessment when encountered. Manipulative and mind-body-based therapies, such as chiropractic and relaxation techniques and homeopathy, have previously been popular among JIA patients [26-29,33]. Here, the use of mind-body and manipulative CAM was rare. Furthermore, there was no significant difference in the reported CAM use between JIA and IBD patients, although the latter group of patients seemed to use CAM more frequently.

For JIA patients, longer disease duration has been associated with CAM use [16]. Interestingly, we found no significant difference in disease duration with respect to CAM use. The type of medication had no association with CAM use and, for example, those on immunosuppression and/or biologics did not report using CAM more frequently, unlike what we had originally hypothesized.

Indicators identified to predict CAM use include a desire for better disease control and the perceived helpfulness of CAM [28,34]. The conventional medications for IBD and JIA patients alleviate symptoms but are often unsatisfactory or have significant side effects. Many drugs are poorly studied in paediatric populations [8], which may evoke concern. Reassuringly, CAM use does not seem to associate with overall reduced adherence to medical therapy [35]. Caregivers reported that the side effects from prescribed medicine, the disappointment with their effects [22] and the wish to relieve their child’s pain and to improve his/her overall wellbeing [27,28] were the major reasons for CAM use. The reasons for the use of CAM were not questioned in our study. Here, according to self-reports, the adherence to conventional medication was nevertheless excellent in more than 70% of the IBD and JIA patients. Thus, we found no significant reduction of treatment compliance among adolescent IBD/JIA patients using CAM.

Many patients or parents may be reluctant to tell their physician about CAM use, possibly thinking that it is not relevant [18,27,36] but this aspect was not assessed in the current study. Furthermore, patients tend to believe that CAM therapies are safer and less toxic than conventional medication, which is not always true. Alarming reports show severe liver failure resulting in the need for transplantation after use of, for example, certain herbal products [19,37]. When taking care of paediatric patients with chronic diseases, every effort should be made to make the families feel confident and willing to share information regarding CAM use. In most cases, the use of CAM may not be especially harmful, but it may be unnecessary for the patients and this also needs to be discussed with the families.

This study assessed for the first time CAM use simultaneously among adolescents with IBD or JIA. The returned questionnaires were generally completed in full. The results were related to disease activity and medication adherence via chart review. The study was mainly conducted by the personnel taking care of the IBD patients, which likely diminished the interest of JIA patients to participate, especially those who received the questionnaires by mail. We did not sent out reminders, as the initial mailing of the questionnaires was done at the end of the school term. The overall response rate was satisfactory, but the smaller proportion of JIA respondents was unexpected. The IBD/JIA population, however, is likely to be representative of the respective patient populations, as the comprehensive care for these patients is provided at our tertiary level unit and similar services are not available elsewhere.

## Conclusions

Physicians should familiarise themselves with the basic concepts of CAM and actively ask their patients about their CAM use, with it being frequent among adolescent IBD and JIA patients. Regular use of CAM associates with dietary restrictions to be detected at medical follow-ups. Reassuringly, adherence to disease modifying drugs is good in young CAM users, but potential pharmacological interaction or the toxicity of certain CAM products warrants greater awareness.

## Competing interests

The authors declare that they have no competing interests.

## Authors’ contributions

PN participated in the study design, performed the primary data analyses and wrote the first draft of the manuscript. LM-S contributed to data collection and the writing of the manuscript. KA contributed to the recruitment of patients with rheumatic disease and the writing of the manuscript. KLK conceived the study and supervised the design, performed the final data analyses and contributed to the writing of the manuscript. All authors approved the final manuscript.

## Pre-publication history

The pre-publication history for this paper can be accessed here:

http://www.biomedcentral.com/1472-6882/14/124/prepub

## Supplementary Material

Additional file 1 QuestionnaireCAM2012.pdf (in Finnish). Click here for file
