# Supplementary material for: Complementary and alternative medicine use in adolescents with inflammatory bowel disease and juvenile idiopathic arthritis
Source: BMC Complement Altern Med. 2014 Apr 4;14:124. doi: 10.1186/1472-6882-14-124 (PMC4101821; doi:10.1186/1472-6882-14-124)
Supplement: Additional file 1 — QuestionnaireCAM2012.pdf (in Finnish). [file 1472-6882-14-124-S1.pdf]

22.5.2012

Kyselykaavake IBD-potilaalle / reumapotilaalle

Ole hyvä ja rastita sopivin vaihtoehto tai täydennä tiedot. Voit halutessasi kirjoittaa lisää kääntöpuolelle tai marginaaliin.

Täytä kaavake kaikessa rauhassa kotonasi, ja **postita oheisella palautuskuorella** *(postimaksu maksettu).*

Täyttöpäivämäärä: ___ / ___ 201__ Kaavakkeen täyttäjä: ____________________________

Potilaan nimi: ____

1. **Syntymäpäivä:** __________________
2. **Paino:** ______ kg
3. **Pituus:** ______ cm
4. **Perheen koko:** ____ 18v. täyttänyttä tai aikuista ____ 7-17 -vuotiasta ____ alle 7-v. (merkitse taloudessanne asuvien lukumäärät)
5. **Asuinpaikkasi väkiluku** (rastita oikea vaihtoehto)**:**

   ___ alle 10 000 ___ 10 000-50 000

   ___ 50 000-100 000 ___ yli 100 000

6. **Asuinpaikkasi postinumero:** _______________________
7. **Asumismuoto** (rastita oikea vaihtoehto)**:**

   ___ omakotitalo ___ rivitalo ___ kerrostalo

8. **Onko perheessänne lemmikkieläimiä** (koira, kissa, jyrsijä, matelija tms.) **tai kodin yhteydessä muita eläimiä** (esim. karjaa, hevosia, tarhaeläimiä)?

   Ei ______ Kyllä______

   Jos vastasit kyllä: Millaisia ja kuinka paljon?

   ____

9. **Oletko viimeisen vuoden aikana noudattanut jotain erityistä ruokavaliota tai välttänyt / joutunut välttämään joitakin ruoka-aineita?**

Ei ______ Kyllä______

Jos vastasit kyllä, erittele mitä ruokavaliota ja miksi?

____

____

10. **Tupakoitko?**

Ei ______ Kyllä,______ savuketta/vrk

11. **Tupakoiko joku perheenjäsenesi?**

Ei ______ Kyllä, sisällä______ Kyllä, ulkona______

12. **Diagnoosisi** (rastita oikea vaihtoehto):

Crohnin tauti ______
haavainen paksusuolentulehdus ______
välimuotoinen koliitti ______
lastenreuma ______
muu, mikä? ____

13. **Säännöllisesti käyttämäsi lääkärin määräämä lääkitys:**

____

____

____

14. **Kuinka usein sinulta keskimäärin unohtuu ottaa lääkärin määräämät reseptilääkkeet?** (rastita oikea vaihtoehto)

Kerran kuukaudessa tai harvemmin ______

Viikoittain ______

Päivittäin ______

15. **Oletko edeltävän 12 kk aikana käyttänyt seuraavia valmisteita**?

*Merkitse mahdollisimman tarkka **valmisteen nimi** ja **määrä** ja **kuinka usein** käytät valmistetta. Jos et ole käyttänyt kyseisen ryhmän valmisteita, rastita "en".*

15.1. ***D-vitamiinivalmiste*** (esim. Minisun, Devitol, Devisol, Elivo tms.)

En ______ Kyllä______, mitä?____

Jos vastasit kyllä, arviolta kuinka usein ja kuinka paljon:

____

15.2. ***kalsiumvalmiste tai kalsium-vitamiinivalmiste*** (esim. Calcichew, Kalsipos, Multi-Kalkki tms.)

En ______ Kyllä______, mitä?____

Jos vastasit kyllä, arviolta kuinka usein ja kuinka paljon:

____

15.3. ***monivitamiinivalmiste tai muuta kuin D-vitamiinia sisältävä valmiste mm. foolihappo-, A-, E, B- tai C-vitamiinivalmiste/yhdistelmä*** (esim. Multi-Tabs, Multivita, Sanasol tms.)

En ______ Kyllä______, mitä?____

Jos vastasit kyllä, arviolta kuinka usein ja kuinka paljon:

____

15.4. ***hivenaine- tai mineraalivalmiste***
(esim. rauta, magnesium, pii, sinkki, seleeni, kromi, mangaani, boori, kupari)

En______ Kyllä______, mitä?____

Jos vastasit kyllä, arviolta kuinka usein ja kuinka paljon:

____

15.5. ***rasvahappo- ja omegaöljy/kalaöljyvalmisteet*** (esim. Möller, Omegat, Lysi tms.)

En______ Kyllä______, mitä?____

Jos vastasit kyllä, arviolta kuinka usein ja kuinka paljon:

____

15.6. ***heravalmisteet*** (esim. Molkosan, Finnmolke tms.)

En______ Kyllä______, mitä?____

Jos vastasit kyllä, arviolta kuinka usein ja kuinka paljon:

____

15.7. ***levävalmisteet ja –uutteet*** (esim. spiruliina tms.)

En______ Kyllä______, mitä?____

Jos vastasit kyllä, arviolta kuinka usein ja kuinka paljon:

____

15.8. ***aminohappovalmisteet*** (esim. leusiini, valiini, arginiini, tyrosiini tms.)

En______ Kyllä______, mitä?____

Jos vastasit kyllä, arviolta kuinka usein ja kuinka paljon:

____

15.9. ***antioksidanttivalmisteet*** (esim. ubikinoni, beetakaroteeni tms.)

En______ Kyllä______, mitä?____

Jos vastasit kyllä, arviolta kuinka usein ja kuinka paljon:

____

15.10. ***aloe vera -valmisteet*** (suun kautta, ei voiteina tai muutoin paikallisesti annosteltuna)

En______ Kyllä______, mitä?____

Jos vastasit kyllä, arviolta kuinka usein ja kuinka paljon:

____

15.11. ***maitohappobakteerivalmisteet jauheina/tabletteina/tippoina*** (esim. Rela, Idoform tms.)

En______ Kyllä______, mitä?____

Jos vastasit kyllä, arviolta kuinka usein ja kuinka paljon:

____

15.12. ***maitohappobakteerivalmisteet elintarvikeina***
(esim. lactobacillus asidophilus, bifidobakteeri, Gefilus-tuotteet tms.)

En______ Kyllä______, mitä?____

Jos vastasit kyllä, arviolta kuinka usein ja kuinka paljon:

____

15.13. ***vatsan toimintaa edistävät valmisteet*** (kuituvalmisteet, pellavansiemen(rouhe), psyllium tms.)

En_______ Kyllä______, mitä?____

Jos vastasit kyllä, arviolta kuinka usein ja kuinka paljon:

____

15.14. ***luontaistuotekaupan kasvi-, yrtti- tai marjavalmisteet***
(esim. tyrni, goji-marjat, punahattu-uute, nokkonen, inkivääri, valkosipuli tms.)

En______ Kyllä______, mitä?____

Jos vastasit kyllä, arviolta kuinka usein ja kuinka paljon:

____

15.15. ***entsyymivalmisteet*** (esim. laktaatsi, lipaasi, amylaasi tms.)

En______ Kyllä______, mitä?____

Jos vastasit kyllä, arviolta kuinka usein ja kuinka paljon:

____

15.16. ***urheilijan lisäravinteet*** (esim.proteiinivalmisteet tms.)

En______ Kyllä______, mitä?____

Jos vastasit kyllä, arviolta kuinka usein ja kuinka paljon:

____

15.17. ***energiajuomat*** (esim. Teho, Battery, ED, Red Bull, energiashotit tms.)

En______ Kyllä______, mitä?____

Jos vastasit kyllä, arviolta kuinka usein ja kuinka paljon:

____

15.18. ***virkistys- ja palautusjuomat***
(esim. Fenix. Hyvää Päivää tms., ei koske tavallisia virvoitusjuomia/limonadeja tai kahvijuomia)

En______ Kyllä______, mitä?____

Jos vastasit kyllä, arviolta kuinka usein ja kuinka paljon:

____

15.19. ***muut lisäravinteet*** (esim. Nutridrink, Resource, Addera tms.)

En______ Kyllä______, mitä?____

Jos vastasit kyllä, arviolta kuinka usein ja kuinka paljon:

____

15.20. ***laihdutus- tai paastovalmisteita***
(esim. detox-valmisteet, paastomehut tai -teet, laihdutusateriat kuten Nutrilett tms.)

En______ Kyllä______, mitä?____

Jos vastasit kyllä, arviolta kuinka usein ja kuinka paljon:

____

15.21. ***kolesterolia alentavia valmisteita elintarvikkeina tai jauheena/tablettina/tippoina***
(esim. Benecol, punariisi tms.)

En______ Kyllä______, mitä?____

Jos vastasit kyllä, arviolta kuinka usein ja kuinka paljon:

____

15.22. ***muuta, mitä?***
Kirjoita tähän myös, jos käytät jotain valmistetta, mutta olet epävarma siitä, mihin ryhmään käyttämäsi valmiste kuuluu.

____

____

Jos vastasit johonkin kohtaan kyllä, muistithan eritellä ***kuinka usein*** *ja* ***millä annoksella*** *käytät valmistetta!*

16. **Oletko <u>edeltävän 12 kk aikana</u> ollut antibioottikuurilla?**

Ei ____ Kyllä_____, milloin ja miksi?____

Valmisteen nimi:____

Lääkekuurin kesto vuorokausina: _______ vrk

17. **Oletko <u>edeltävän 12 kk aikana</u> saanut seuraavia hoitoja:**
(rastita oikea vaihtoehto)

17.1.	hypnoosi/hypnoterapia	Kyllä______	Ei______
17.2.	vyöhyketerapia	Kyllä______	Ei______
17.3.	akupunktio	Kyllä______	Ei______
17.4.	reiki-hoidot, kuhne, kuppaus	Kyllä______	Ei______
17.5.	fysikaalista hoitoa	Kyllä______	Ei______
17.6.	muuta, mitä?		

____

____

____

Tutkijalääkäri saa ( ) ei saa ( ) ottaa minuun tarvittaessa yhteyttä, jos ilmenee tarvetta tietojen tarkentamiseen tähän tutkimukseen liittyen.

Puhelinnumero, josta tavoittaa päivisin: ____

Kiitos vaivannäöstäsi!

Pauliina Nousiainen
Tutkijalääkäri/lastentautien erikoislääkäri
KYS Lasten ja nuorten klinikka, Kuopio
PL1777, 70211 Kuopio

puh: 050 329 3923
pauliina.nousiainen@kuh.fi
